# The Challenge of Managing Refractory Psychosis Amid Multiple Medication Side Effects: A Case Report and Review of the Literature

**DOI:** 10.7759/cureus.50063

**Published:** 2023-12-06

**Authors:** Alejandro Rodulfo, Sabina Goldstein, Zina Meriden

**Affiliations:** 1 Psychiatry, Memorial Regional Hospital, Hollywood, USA

**Keywords:** treatment-resistant schizophrenia, clozapine, sialorrhea, akathisia, hyperprolactinemia, tardive dyskinesia, antipsychotics

## Abstract

Antipsychotics are the mainstay for the treatment of schizophrenia and other psychotic disorders; however, these agents are associated with an extensive side effect profile that may complicate treatment outcomes. We present the case of a 35-year-old woman with a history of schizoaffective disorder and five prior psychiatric hospitalizations.

The patient first presented to the hospital for disorganized behavior, in addition to poor sleep, auditory hallucinations, and racing thoughts in the context of medication nonadherence. She received two loading doses of intra-muscular paliperidone with fair symptomatic improvement. After discharge, she was scheduled to receive a monthly dose of paliperidone, which she missed, resulting in decompensation, re-emergence of psychosis, and another hospitalization two months later. She was given the missed dose with no improvement and progressive deterioration, for which alternative agents were tried. She received olanzapine and was tried briefly on quetiapine and haloperidol as well, with no benefit, and she also developed abnormal perioral movements. She was reloaded with paliperidone, and her psychotic symptoms improved, although she developed akathisia and hyperprolactinemia. The patient returned to the hospital two days later after being discharged, due to disorganized behavior and multiple delusions. Clozapine was started and titrated to 100 mg qam and 200 mg qhs. While on clozapine, she developed profuse sialorrhea that was treated with sublingual atropine drops, and by the time of discharge psychotic symptoms had markedly improved, perioral movements diminished, and prolactin level trended down. The patient maintained stability for over a year after the last admission.

Identifying antipsychotics to successfully treat refractory psychosis and managing their multiple potential side effects is challenging but can result in better quality of life for patients as well as improved treatment adherence. This case report is unique in the way it illustrates this point, while discussing different approaches to managing multiple side effects that can happen simultaneously.

## Introduction

Schizophrenia is a psychiatric illness that can present with positive, negative, and cognitive symptoms and often leads to functional impairment in affected patients [1]. A significant number of patients with schizophrenia do not fully respond to antipsychotic medication and are therefore considered to have treatment-resistant schizophrenia (TRS). The exact definition of TRS varies among psychiatrists, but the consensus is that, at a minimum, a patient must have persistent psychotic symptoms despite an adequate trial of at least two antipsychotic medications. Howes et al. defined an adequate trial as at least six weeks of treatment with an antipsychotic medication at a therapeutic dose (defined as a minimum of the equivalent of 600 mg chlorpromazine), and, particularly for oral medications, at least 80% compliance over the course of 12 weeks. Furthermore, at least one of the antipsychotic medications should be a second-generation antipsychotic [2]. Studies on TRS highlight the importance of relying on an objective measurement of symptom severity to determine if a patient truly failed an antipsychotic medication, such as the Positive Scale, Negative Scale, and General Psychopathology Scale (PANSS) [3].

Because patients who have TRS have already failed at least two antipsychotics, the treatment for TRS is often limited. Clozapine is the only FDA-approved pharmacologic treatment for TRS. Multiple studies, including the CATIE trial and CUTLASS, have illustrated the effectiveness of clozapine for patients with TRS. Still, nearly one-third of patients are clozapine-resistant. Augmenting clozapine treatment with an additional psychotropic medication, including another antipsychotic, an antidepressant, a mood stabilizer, or a glutamatergic agent, has shown limited success. Numerous studies and metanalyses provide evidence that electroconvulsive therapy (ECT) may be beneficial for patients with clozapine-resistant TRS and that patients may benefit from the synergistic effect of receiving ECT as an adjunct to clozapine [4]. Despite the proven efficacy of clozapine, it is often utilized as a last-resort medication due to the potential side effects, the frequent blood work necessary for monitoring patients, and the requirement that prescribers to be registered in clozapine REMS [5].

Some of the side effects that can be encountered more often in patients taking clozapine compared to other antipsychotics include myocarditis, neutropenia, or agranulocytosis, but they are still rare. Sialorrhea on the other hand is much more common. Clozapine, like most antipsychotic medications, can be associated with a myriad of other side effects as well. These can be metabolic (weight gain, hypertension, adverse effects on lipid and glucose metabolism) and anticholinergic (dry mouth, constipation, blurred vision, mydriasis, and tachycardia), as well as orthostatic hypotension, neuroleptic malignant syndrome, QT prolongation, and sudden cardiac death. The potential for extrapyramidal side effects (akathisia, parkinsonism, dystonia) is much lower with clozapine compared to other antipsychotics [6]. Among patients with schizophrenia, medication side effects are highly prevalent and significantly associated with medication nonadherence, which is significantly associated with increased use of healthcare resources. Prevention, identification, and effective management of medication-induced side effects are important to maximize treatment adherence and improve clinical outcomes such as reduced morbidity and mortality and decreased utilization of hospital services [7].

## Case presentation

The patient is a 35-year-old Hispanic female, with a past psychiatric history of schizoaffective disorder, bipolar subtype, with five prior psychiatric hospitalizations, and seemingly adequate trials of haloperidol, paliperidone, quetiapine, cariprazine, divalproex, lamotrigine, and escitalopram (doses and duration unknown), with reported limited adherence to psychiatric medications in the outpatient setting. She had three consecutive psychiatric hospitalizations for acute psychosis under our care at a community-based hospital in South Florida, from September to December of 2019, during which psychotic symptoms proved refractory to different antipsychotics. During that time, the patient developed the following side effects: tardive dyskinesia (TD), akathisia, hyperprolactinemia, and sialorrhea, all of which were addressed with different pharmacological interventions. She was tried on paliperidone, haloperidol, quetiapine, and olanzapine and was eventually stabilized on clozapine monotherapy, maintaining stability without any re-admissions one year after discharge.

Background history

There was a family history of schizophrenia versus bipolar disorder in the paternal grandmother and two brothers with bipolar disorder. She was born and raised by both biological parents in the Dominican Republic, had five siblings (four brothers and one sister), and moved to the United States at the age of 8. She had three sons aged 14, 12, and 7 and lived with them and her fiancé. She obtained a GED diploma and had no history of learning disability or developmental delay. She was collecting disability as she had not been working for the past four years due to her severe mental illness but had recently started working again part-time in customer service for a credit card company. She was asked to cover additional hours for another employee, resulting in the patient working more hours/week than anticipated. This and the stress of taking care of her three children at home were factors leading to psychiatric decompensation. Per family, she was reportedly diagnosed with schizophrenia versus bipolar disorder at the age of 16. Per review of available medical records, she had been psychiatrically hospitalized twice in our hospital back in 2016 for psychosis. On the first admission, she presented with auditory hallucinations, agitation, and bizarre behavior and was discharged on oral paliperidone and lorazepam. On the second admission, she presented after lighting a paper on fire and threatening to burn her house. She was discharged on quetiapine and divalproex. She was seen once in 2014 after her significant other found her lying on the street and crawling under a car. She was also seen in 2017 after being found wandering the premises of her children's school and becoming combative when approached by a law enforcement officer. It is unknown to us if she met the criteria for a major mood episode at the time of these hospitalizations. In 2014 and 2017, she was transferred from the emergency department to another psychiatric hospital in the community and the treatment course there is unknown. The patient followed up with an outpatient psychiatrist at a community behavioral clinic every 2 to 3 months and was intermittently adherent to psychotropic medications, with reported periods of stability while taking them regularly although we were unable to confirm this. Past and current history of substance use disorder was denied. Past medical history was pertinent for a motor vehicle accident in 2012 in which she suffered a 12 cm avulsion to her right upper extremity, a subtle right tibial plateau fracture, and spleen and liver lacerations, with no acute findings on computerized tomography of the brain without contrast.

Diagnostic assessment and hospital course

The patient was initially admitted to the hospital involuntarily via law enforcement for gross psychosis in the form of disorganized behavior and auditory hallucinations. This had been preceded by a four-day long period of decreased sleep, in the context of medication noncompliance and having lost her new job. Using the Diagnostic and Statistical Manual of Mental Disorders (DSM-5) criteria, a diagnosis of schizoaffective disorder was made (295.70) [8]. Given documented prior positive response to paliperidone, she was initiated on oral paliperidone which was transitioned to the long-acting-injectable (LAI) paliperidone palmitate to address the issue of medication noncompliance. She received two intramuscular (IM) loading doses during the first admission (234 and 156 mg respectively) with fair symptomatic improvement. After discharge, she was scheduled to receive a monthly dose of paliperidone 117 mg (corresponding dose for oral paliperidone 6 mg, the dose the patient was on) at our hospital’s long-acting-injection clinic, but she did not follow up, resulting in decompensation and re-emergence of psychotic symptoms. She presented again two months later, involuntarily and via a law enforcement officer, for auditory hallucinations, disorganized behavior, and delusions that her children were kidnapped which were preceded by episodes of impulsive behavior (shopping sprees). On arrival at the emergency room, she was agitated and appeared to be responding to internal stimuli, for which she was re-admitted to the hospital. The missed dose of IM paliperidone palmitate was administered (at a dose of 156 mg instead given the clinician's judgment that 117 mg would be subtherapeutic), but this time she did not exhibit meaningful improvement, requiring multiple daily as needed doses of other antipsychotics for the treatment of agitation. Initiation of valproic acid or lithium was considered at this time for mood stabilization and as an adjunctive treatment for refractory symptoms but given significant history of noncompliance in the outpatient setting, stabilization with antipsychotic therapy only was pursued. She was re-loaded with two doses of paliperidone palmitate LAI (234 mg and 156 mg), given prior response to this antipsychotic. Due to the severity of her presentation, it was deemed that the risk of administering a supratherapeutic dose of the medication outweighed the risks. This time, in contrast to her response in the past, no significant improvement was noted.

A trial of quetiapine for five days in total followed, titrated to a dose of 600 mg daily (in addition to additional doses of IM olanzapine and/or haloperidol for the treatment of acute agitation). But these were unsuccessful as well. The patient continued to exhibit bizarre behavior, delusional thoughts of religious and persecutory nature, ideas of reference, Capgras delusions, and self-neglect while on the psychiatric unit. Additionally, the patient developed abnormal perioral movements concerning the onset of TD, likely related to antipsychotic polypharmacy and high doses. Decision was made to do a 48-hour “washout” in which no oral or IM antipsychotics were given. The main concerns at the time were worsening of the aforementioned side effects and the possible development of neuroleptic malignant syndrome due to multiple antipsychotic medications at high doses. Benztropine was initiated for prevention of extrapyramidal side effects.

At this time, both a clozapine trial and ECT were considered. However, the team was concerned she would not adhere to weekly CBCs that are required with clozapine. There was no access to ECT at our center, and the patient’s parents declined a transfer to a different facility. Following the completion of the oral antipsychotic washout, the patient exhibited fair improvement of psychotic symptoms as evidenced during serial hospital assessments for an additional 48 hours. During this time, the patient developed akathisia, which was managed with propranolol with good response. During this second hospitalization as well, a prolactin level was obtained and found to be significantly elevated at 358 ng/mL (normal < 25 ng/ml in nonpregnant women). The test was ordered based on clinical judgment from the covering provider, as there was not an overt indication at the time. Magnetic resonance imaging of the brain without contrast was obtained to rule out a prolactinoma, and it was nondiagnostic for acute abnormalities or any space-occupying lesion. TSH, Syphilis antibody, HIV, homocysteine level, tissue transglutaminase IgG, vitamin B12, and folate level were within normal limits. As the patient's psychosis was refractory to other medications but responded to paliperidone, and she had no evident signs or symptoms of hyperprolactinemia, she was discharged with a plan to receive monthly IM paliperidone and follow-up with endocrinology as an outpatient. An overview and timeline of relevant events (including side effects and diagnostic tests), medications, and comments pertaining to the initial two psychiatric hospitalizations are presented in Table 1.

**Table 1:** Timeline of events, medications, and comments related to the first and second psychiatric hospitalizations IM: Intramuscular

Date	Events and medications	Comments
9/18/19	First psychiatric hospitalization	Disorganized behavior and auditory hallucinations that were preceded by decreased sleep
9/20/19	Paliperidone palmitate 234 mg	n/a
9/24/19	Paliperidone palmitate 156 mg	Discharged home afterward, with plan to receive monthly injection of paliperidone palmitate 117 mg
11/04/19	Second psychiatric hospitalization	Auditory hallucinations, disorganized behavior, and nonbizarre delusions preceded by episodes of impulsive behavior
11/05/19	Paliperidone palmitate 156 mg	“Catch up” dose between the six weeks as per the medication’s package insert (higher dose than she was to receive as outpatient)
11/08/19	Paliperidone palmitate 234 mg	In addition to oral paliperidone 9 mg daily, this was a supratherapeutic dose, it was deemed that the potential benefits outweighed the risks given the severity of the patient's presentation
11/11/19	Paliperidone palmitate 156 mg	n/a
11/09/19 – 11/14/19	Trial of quetiapine lasting five days	Titrated to a maximum dose of 600 mg daily before discontinuation
11/14/19	Patient developed abnormal perioral oral movements, treated with benztropine 1 mg twice daily	Concern for onset of TD, and possible development of NMS led to deciding discontinuing treatment with additional oral and IM as needed antipsychotics, and employing lorazepam for the treatment of agitation instead
11/15/19	Akathisia was noted, and treatment with propranolol 10 mg three times a day was started	Significant improvement was noted after 24 hours
11/18/19	Discharged after completing an antipsychotic “washout”, and showing fair improvement in symptoms	Discharged with plan to receive paliperidone palmitate 234 mg IM monthly injections

Unfortunately, she returned just two days later, again involuntarily, brought in by law enforcement officers, after exhibiting disorganized behavior and presenting with bizarre and paranoid delusions. At this point, the decision was made to initiate clozapine for TRS after extensive conversation with the patient’s parents, which was titrated to a final dose of 100 mg qam and 200 mg qhs over the course of two weeks, with a corresponding therapeutic level of 353 ng/mL. While on clozapine, she developed profuse sialorrhea that did not respond to glycopyrrolate but resolved with sublingual atropine drops. By the time of discharge, her psychotic symptoms had markedly improved, perioral movements had diminished, and her prolactin level trended down to 338 ng/mL. She was placed on a waiting list for transfer to a local state hospital, but given clinical improvement, she was able to return with family and resume work while maintaining stability on clozapine over one year after discharge with no repeat hospitalizations. An overview and timeline of relevant events (including side effects and diagnostic tests), medications, and comments pertaining to the third psychiatric hospitalization and one-year follow-up are presented in Table 2.

**Table 2:** Timeline of events, medications, and comments related to the third psychiatric hospitalization and one-year follow-up

Date	Events and medications	Comments
11/20/19	Third admission to the psychiatric hospital	Disorganized behavior, bizarre and paranoid delusions
11/21/19	Initiated clozapine titration protocol	Achieved final dose of 100 mg qam and 200 mg qhs on 12/05/19
11/27/19	Initiated petition to the local state hospital	The waiting list was about 2-3 months in the state of Florida at the time the patient was treated
12/09/19	Patient developed profuse sialorrhea. Initiated on glycopyrrolate 1 mg TID	Patient had limited response to this anticholinergic agent
12/12/19	Clozapine level was collected	Therapeutic level of 353 ng/mL
12/24/19	Switched glycopyrrolate to SL atropine drops, 2 drops TID	Significant improvement in sialorrhea noted after 24 hours
12/26/19	Last discharge from the hospital on Clozapine 100 mg qam / 200 mg qhs. Patient had exhibited significant improvement for the last 72 hours	Discharged home and removed from waiting list to the state hospital, per family members request, given resolution of gross psychosis
January 2021	Patient was contacted via phone and reported ongoing stability (living with family and holding a job) on same regimen of clozapine	Patient followed up at a local psychiatric community clinic, with no re-admissions or ER visits at our hospital as of August 2022

## Discussion

This case presentation goal is to bring attention to clinicians that the challenge of managing multiple co-occurring antipsychotic side effects can occur in the setting of treatment-resistant psychosis, and both should be addressed simultaneously by weighing the risks and benefits, including the imminent threat posed by untreated gross psychosis or a life-threatening side effect. We present a brief discussion of the side effects experienced by our patient. 

TD is proposed to stem from dopamine receptor super-sensitivity and is more frequently seen in patients on long-term antipsychotic treatment (several years to decades). It is more commonly seen with potent dopamine antagonists such as haloperidol or fluphenazine. Lowering the dose of antipsychotic medications, switching to an atypical antipsychotic or the use of anticholinergic agents are common strategies to manage, but in many cases, this side effect can be irreversible even years after the responsible agent is stopped. Back in 2017, the first vesicular monoamine transporter 2 (VMAT2) inhibitors, valbenazine, and deutetrabenazine received FDA approval for the treatment of TD. These medications work by causing a depletion of neuroactive peptides such as dopamine in nerve terminals; most clinicians don’t have significant experience with these medications, which were not readily available in most community hospital settings at the time this patient was treated. Although benztropine was used with this particular patient when there was concern for TD, there is no major evidence in the literature to suggest its efficacy in treating this condition [9]. Access and adherence to VMAT2 inhibitors may be limited by insurance coverage and regulatory issues, but evidence shows that these are effective and safe in most patients [10]. 

Akathisia is a state of agitation, distress, and restlessness that is an occasional side-effect of antipsychotic and antidepressant drugs [11]. Centrally acting beta-blockers such as propranolol are first-line treatments for akathisia as anticholinergic medications have a more limited impact on this side effect. When these are contraindicated (for example in a patient with hypotension or bradycardia) benzodiazepines can be used as well as a second line with good effect [12].

Hyperprolactinemia, which is another common side effect of antipsychotics, frequently goes unrecognized by clinicians. As of today, there are no guidelines indicating when to check a prolactin level, or if it should be obtained at baseline [13]. Clinical presentation, such as the development of galactorrhea, for example, should guide consideration of checking the level, additional endocrine workup, and adjustment of antipsychotic treatment. Switching to a second-generation / prolactin-sparing agent such as clozapine, quetiapine, ziprasidone, or olanzapine is a good strategy to manage this side effect. However, switching medications may not be clinically feasible or advisable in some cases. For example, switching patients who have been stable for a considerable period on a certain medication, poses the risk of decompensation. Adding aripiprazole, which is an antipsychotic with partial agonism at the D2 receptors, is another useful strategy that can help lower the prolactin level. The addition of cabergoline or bromocriptine can also be considered, but these agents can potentially exacerbate psychotic symptoms given their direct agonism at the D2 receptor [14,15].

Sialorrhea is a side effect seen with different antipsychotic medications (although the dry mouth is more common given the anticholinergic profile from most of these); however clozapine-induced-sialorrhea is quite frequent (present in over 90% of patients on this medication to some degree), as its metabolite norclozapine exerts a potent cholinergic agonist effect at the salivary glands, and in most patients tends to be more prominent at nighttime, but it can also be present during the day which can be quite stigmatizing and a potential reason for nonadherence [16]. Sublingual atropine drops are the first-line treatment and are very well tolerated as there is no significant systemic absorption. Every three months botulinum toxin injections are also effective and can assist with decreasing medication burden. Systemic anticholinergic agents such as glycopyrrolate, trihexyphenidyl, or benztropine are third-line given their significant side effect profile [17].

Switching antipsychotic medications to address side effects can be the best intervention in most clinical scenarios. When refractory psychosis is part of the picture, a trial of clozapine should be considered, as this carries the advantage of a decreased risk for EPS, TD, and hyperprolactinemia. Unfortunately, clozapine carries its own risks for other side effects, though for the most part, these can be addressed [18]. This case in particular teaches that the early consideration of clozapine can be the simplest intervention to prevent the onset of side effects more closely related to potent D2 antagonists. When continuing to increase the dose of antipsychotics to supratherapeutic ranges, and combining multiple agents to treat refractory symptoms, the risk for side effects increases, the reason for which these clinical practices should be minimized as much as possible. With our patient it could have decreased, or even prevented, some of the side effects that she experienced, making her case more generalizable to the general population. The introduction of agents with long-acting formulations further complicates the clinical picture in these cases, especially when used in supratherapeutic doses, as the prolonged release of medication makes it impossible to do a meaningful washout period when cross-titrating agents, as it was seen in this case. Similarly, the time constraints associated with the scrutiny of the length of stay in inpatient psychiatric units, as well as more rapid medication adjustments can lead to shorter trials in which agents are switched before an appropriate trial is completed, leading to increased risk of polypharmacy and side effects. 

## Conclusions

Identifying antipsychotics to successfully treat refractory psychosis and managing their multiple potential side effects can be challenging. Side effects can be addressed by lowering the medication dose or adjusting their dosing schedule, a change of antipsychotic, or adding concomitant medications. Treatment-resistant schizophrenia should warrant a trial of clozapine, an agent known to have lower association with TD, EPS, and hyperprolactinemia; however, it is not exempt from its own significant and potentially life-threatening side effect profile that is beyond the scope of this report. Appropriate length of medication trials while minimizing supratherapeutic dosing and antipsychotic polypharmacy is another important consideration to prevent side effects from these medications, something that is well illustrated in this case report. It is very important for psychiatrists to give special consideration to addressing medication side effects in their patients, as when not treated they can often lead to treatment nonadherence and poor clinical outcomes.
